# Procalcitonin is not an independent predictor of 30-day mortality, albeit predicts pneumonia severity in patients with pneumonia acquired outside the hospital

**DOI:** 10.1186/s12877-018-1008-8

**Published:** 2019-01-07

**Authors:** Takanori Akagi, Nobuhiko Nagata, Hiroyuki Miyazaki, Taishi Harada, Satoshi Takeda, Yuji Yoshida, Kenji Wada, Masaki Fujita, Kentaro Watanabe

**Affiliations:** 1 Department of Respiratory Medicine, Fukuoka University Chikushi Hospital, 1-1-1 Zokumyoin, Chikushino-city, 818-8502 Japan; 2 Department of Respiratory Medicine, Faculty of Medicine, Fukuoka University, Fukuoka-city, 814-0180 Japan

**Keywords:** Procalcitonin, C-reactive protein, Pneumonia, Pneumonia severity, Prognosis, Elderly

## Abstract

**Background:**

Procalcitonin (PCT) is a useful marker for pneumonia. However, its clinical usefulness in elderly patients has not been studied extensively. This study aimed to assess the relationship between PCT and prognosis and pneumonia severity in elderly patients with pneumonia acquired outside the hospital.

**Methods:**

Data considered relevant to pneumonia severity and prognosis were retrospectively obtained from clinical charts of all patients with pneumonia who were admitted to our hospital from 2010 to 2017. The primary outcome was 30-day mortality in elderly patients (aged ≥75 years), and the relationship between PCT levels and pneumonia severity, as determined by the pneumonia severity index (PSI) was also examined.

**Results:**

Data were collected from 667 patients, of which 436 were elderly patients. Multivariate and receiver operating characteristic curve analysis revealed that albumin, body mass index, and PSI class rather than PCT are important factors related to 30-day mortality in elderly patients. PCT was also not an independent prognostic factor in younger patients. PCT levels significantly differed by pneumonia severity (mild, moderate, and severe) in both younger (*p* < 0.001) and elderly (*p* < 0.0001) patients, with levels increasing as severity increased. In contrast, C-reactive protein (CRP) levels and white blood cell counts did not significantly differ by pneumonia severity in younger and elderly patients. A subgroup analysis focused on *Streptococcus pneumoniae* pneumonia revealed that PCT levels differed by severity in elderly patients (*p* = 0.03), with levels increasing as severity increased.

**Conclusion:**

PCT was not an independent predictor of 30-day mortality in both of elderly and younger patients. PCT levels, but not CRP levels, significantly increased with increasing pneumonia severity in younger and elderly patients, although the degree of increase tended to be lower in elderly patients compared to younger patients for the same severity. PCT levels also significantly increased with increasing pneumonia severity in elderly patients with *Streptococcus pneumoniae* pneumonia.

## Background

Community-acquired pneumonia (CAP) is a prominent cause of morbidity and mortality throughout the world [1]. Procalcitonin (PCT) can be used as a marker to differentiate bacterial infection from other non-bacterial infections or non-infectious inflammation [2–4]. Many investigators have reported the clinical usefulness of PCT for assessing the severity of pneumonia or pathogen involved, and for guiding antibiotic use [5–7]. Elderly patients, although representing the large majority of cases of CAP admitted to acute-care hospital wards, are often excluded from clinical studies due to the possible presence of multiple confounders. Although pneumonia occurs mainly in the elderly, the usefulness or clinical significance of PCT in these patients has not been studied extensively. While some studies have reported that PCT levels do not elevate according to the severity in elderly patients [8, 9], others have argued that PCT is useful for predicting the severity of CAP [10, 11].

To date, many studies have reported various prognostic factors for CAP, including the pneumonia severity index (PSI) [12–16], PCT [6, 17–26], albumin [27–30], body mass index (BMI) [27, 31], and brain natriuretic peptide (BNP) or N-terminal-proBNP (NT-proBNP) [32, 33]. However, only a few studies have assessed the role of PCT as a prognostic factor in elderly pneumonia patients. The present study aimed to assess the relationship between PCT levels and 30-day mortality and pneumonia severity in elderly patients (aged ≥75 years) with pneumonia acquired outside the hospital.

## Methods

Clinical charts of all consecutive patients admitted to our hospital from October 2010 to July 2017 with primary diagnoses of CAP and healthcare-associated pneumonia (HCAP) were retrospectively reviewed. We enrolled patients in the study with the same method as we previously reported [34], except for those with HCAP. HCAP included any patients who (1) were hospitalized in an acute care hospital for two or more days within the past 90 days, (2) resided in a nursing home or long-term care facility, or (3) continuously visited a hospital or hemodialysis clinic for intravenous antibiotic therapy, chemotherapy, or hemodialysis [35]. Data on admission considered related to pneumonia severity and prognosis were examined, including age, sex, category of pneumonia occurring outside the hospital (CAP or HCAP), PSI class, comorbidities, BMI, serum albumin, C-reactive protein (CRP), PCT and blood urea nitrogen (BUN) levels, and white blood cell (WBC) count. As for comorbidities, Charlson comorbidity index [36] was also obtained.

Serum PCT levels were determined by chemiluminescence enzyme immunoassay (SphereLight BRAHMS PCT, Wako Pure Chemical Industries, Ltd., Osaka, Japan). The PCT assay has a detection limit of 0.05 ng/mL. When serum PCT levels were under the detection limit, they were assigned a value of 0.05 ng/mL. Serum albumin, CRP and BUN levels were measured with improved bromocresol purple method, latex-turbidimetry and enzymatic method (urease-glutamate dehydrogenase), respectively, using auto-analyzer BioMajesty™ JCA-BM6050 (japan electron optics laboratory co ltd, Tokyo, Japan).

Microbiologic examinations performed were the same as our previous study [34].

The primary outcome was the relationship between PCT levels and 30-day mortality. In addition, we also investigated the relationship between PCT levels and pneumonia severity determined by PSI in younger (age ≤ 74 years) and elderly (age ≥ 75 years) patients, respectively. Pneumonia severity was determined by PSI as follows: PSI class I – III = mild, PSI class IV = moderate, PSI class V = severe. We also compared PCT levels for each pneumonia severity between younger and elderly patients.

At first, we checked normality of data distribution using Shapiro-Wilk test, which revealed that all data examined did not show normal distribution. Therefore, data were expressed as median (1st quartile, 3rd quartile). Relationship between PCT levels and 30-day mortality was examined using logistic regression and receiver operating characteristic (ROC) curve analysis. Variables with *p* < 0.05 in univariate analysis were further examined with multivariate analysis. Differences in PCT levels, CRP levels and WBC counts between younger and elderly patients, and among each pneumonia severity, were analyzed with the Mann-Whitney U test and Kruskal-Wallis test, respectively. When the Kruskal-Wallis test was significant, differences in PCT levels, CRP levels and WBC counts between each pneumonia severity were analyzed with the Dunn test. Comparisons of each category between younger and elderly groups were analyzed with the Mann-Whitney U and chi-square test. Statistical analysis was performed using Excel Tokei 2015 (Social Survey Research Information, Co., Ltd., Tokyo, Japan). *P* < 0.05 was considered statistically significant.

This human study was performed in accordance with the Declaration of Helsinki, and was approved by the Fukuoka University Medical Ethics Review Board (approval: R17–042). The review board exempted the acquisition of informed consent from patients included in the study.

## Results

### Study population

We identified 682 patients with a primary diagnosis of pneumonia during the study period. Of these, 15 with hospital-acquired pneumonia were excluded. The final study population consisted of 667 patients. Clinical features of these patients are summarized in Table 1. The study population consisted of 231 younger patients (younger group) and 436 elderly patients (elderly group). Eleven (4.8%) and 55 (12.6%) patients died from pneumonia within 30 days of admission in the younger and elderly groups, respectively (*p* < 0.01). PCT was measured at the time of admission in 226 (97.8%) and 426 (97.7%) patients of younger and elderly groups, respectively. The ratio of HCAP, PSI class, albumin, PCT, CRP, BUN, and Charlson comorbidity index were significantly different between the younger and elderly groups. As for causative pathogens, *Streptococcus pneumoniae* was the leading pathogen in both of younger and elderly groups. *Klebsiella pneumoniae and Escherichia coli* were more frequent in the elderly group, while *Mycoplasma pneumoniae was more frequent in the younger group.*

**Table 1:** Baseline characteristics

	Total	< 74 years	> 75 years	*P* value
No. of patients	667	231	436	
died within 30 days	66 (10.0%)	11 (4.8%)	55 (12.6%)	< 0.01*
Male / female	424 (63.6%)/243 (36.4%)	1 52 (65.8%)/79 (34.2%)	272 (62.4%)/164 (37.6%)	0.38
Age(years)	80.0 (71.0–86.0)	67.0 (55.0–71.0)	85.0 (80.0–89.0)	< 0.01*
CAP / HCAP	396 (59.4%)/271 (40.6%)	1 75 (75.8%)/56 (24.2%)	221 (50.7%)/21 5 (49.3%)	< 0.01*
PSI class				
I	35 (5.3%)	35 (15.1%)	0	< 0.01*
II	84 (1 2.6%)	75 (32.5%)	9 (2.1%)	
III	151 (22.6%)	50 (21.6%)	101 (23.1%)	
IV	288 (43.2%)	60 (26.0%)	228 (52.3%)	
V	109 (16.3%)	11 (4.8%)	98 (22.4%)	
BMI(kg/m^2^)	19.8 (17.0–22.5)	20.0 (17.9–22.7)	19.6 (16.9–22.5)	0.10
Albumin(g/dL)	3.2 (2.8–3.6)	3.4 (2.9–3.8)	3.1 (2.7–3.5)	< 0.01*
Procalcitonin(ng/mL)	0.35 (0.10–2.09)	0.20 (0.07–0.84)	0.52 (0.13–2.66)	< 0.01*
WBC(/mm^3^)	10,500 (7700–14,400)	11,100 (7600–14,800)	10,300 (7700–13,900)	0.38
CRP(mg/dL)	9.09 (3.99–16.20)	9.91 (4.43–18.0)	8.01 (3.52–15.01)	< 0.01*
BUN(mg/dL)	18 (14–26)	14 (10–19)	20 (15–29)	< 0.01*
Comorbidity Charlson comorbidity index				
0	224 (33.6%)	104 (45.0%)	120 (27.5%)	< 0.01*
1	244 (36.6%)	62 (26.9%)	182 (41.7%)	
2	126 (18.9%)	46 (19.9%)	80 (18.4%)	
3	56 (8.4%)	1 6 (6.9%)	40 (9.2%)	
4	13 (1.9%)	2 (0.9%)	11 (2.5%)	
5	1 (0.1%)	0 (0%)	1 (0.2%)	
6	3 (0.5%)	1 (0.4%)	2 (0.5%)	
Chronic lung disease	1 89 (28.3%)	69 (29.9%)	120 (27.5%)	0.53
Diabetes mellitus	128 (19.2%)	44 (19.0%)	84 (19.3%)	1.00
Dementia	123 (18.4%)	10 (4.3%)	113 (25.9%)	< 0.01*
Cerebrovascular disease	67 (10.0%)	26 (11.3%)	41 (9.4%)	0.50
Chronic heart failure	62 (9.3%)	7 (3.0%)	55 (12.6%)	< 0.01*
Malignancy	33 (4.9%)	9 (3.9%)	24 (5.5%)	0.45
Kidney disease	29 (4.3%)	6 (2.6%)	23 (5.3%)	0.12
Liver disease	15 (2.2%)	3 (1.3%)	12 (2.8%)	0.28
Pathogen Streptococcus pneumoniae	126 (18.9%)	39 (16.9%)	87 (20.0%)	0.35
Haemophilus influenzae	40 (6.0%)	14 (6.1%)	26 (6.0%)	1.00
*Klebsiella pneumoniae*	30 (4.5%)	5 (2.2%)	25 (5.7%)	0.04*
*Escherichia coli*	25 (3.7%)	3 (1.3%)	22 (5.0%)	0.01*
Pseudomonas aeruginosa	23 (3.4%)	10 (4.3%)	13 (3.0%)	0.38
Moraxella catarrhalis	16 (2.3%)	6 (2.6%)	10 (2.3%)	0.78
MSSA	13 (1.9%)	2 (0.9%)	11 (2.5%)	0.24
MRSA	11 (1.6%)	2 (0.9%)	9 (2.1%)	0.35
Serratia marcescens	8 (1.2%)	4 (1.7%)	4 (0.9%)	0.46
Enterococcus faecalis	7 (1.0%	2 (0.9%)	5 (1.1%)	1.00
Stenotrophomonas maltophilia	6 (0.9%)	2 (0.9%)	4 (0.9%)	1.00
Legionella pneumoniae	5 (0.7%)	3 (1.3%)	2 (0.5%)	0.35
Proteus merabilis	5 (0.7%)	1 (0.4%)	4 (0.9%)	0.66
*Enterobacter cloacae*	4 (0.6%)	1 (0.4%)	3 (0.7%)	0.66
Mycoplasma pneumoniae	30 (4.5%)	21 (9.1%)	9 (2.1%)	< 0.01*
Chlamydophila pneumoniae	22 (3.3%)	10 (4.3%)	12 (2.8%)	0.36

*CAP* Community acquired pneumonia, *HCAP* Healthcare-associated pneumonia, *PSI* Pneumonia severity index, *BMI* Body mass index, *WBC* White blood cell, *CRP* C-reactive protein, *BUN* Blood urea nitrogen, *MSSA* Methicillin-sensitive Staphylococccusaureus, *MRSA* Methicillin-resistant *Staphylococcus aureus* Data are presented as number (%) or median (1st quartile - 3rd quartile)

P value: < 74 years vs > 75 years * Statistically significant

### Factors associated with 30-day mortality

**Fig. 1:**
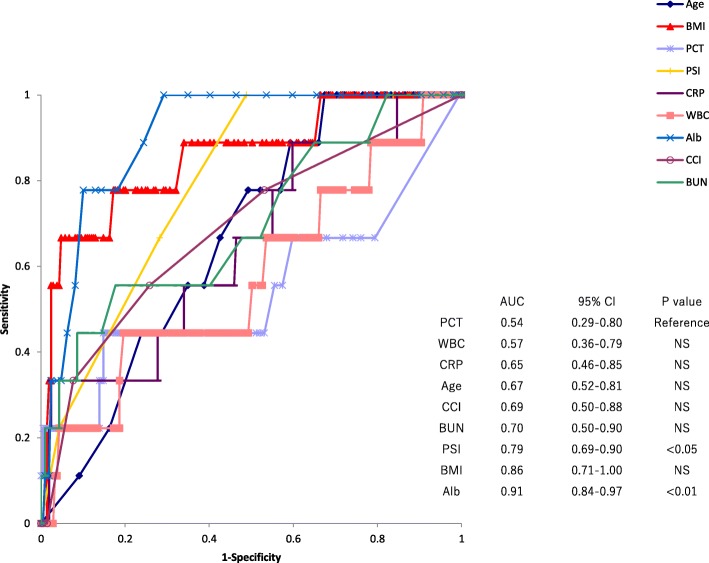
Receiver operating characteristic curve of procalcitonin (PCT) levels, white blood cell (WBC) counts, C-reactive protein (CRP) levels, age, Charlson comorbidity index (CCI), blood urea nitrogen (BUN), pneumonia severity index (PSI) class, body mass index (BMI), and albumin levels for prediction of 30-day mortality of patients younger than 74 years

**Fig. 2:**
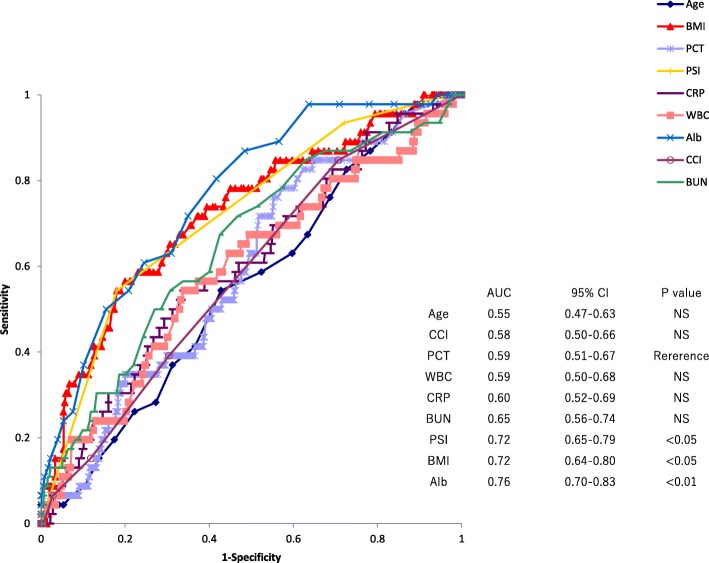
Receiver operating characteristic curve of age, Charlson comorbidity index (CCI), procalcitonin (PCT) levels, white blood cell (WBC) counts, C-reactive protein (CRP) levels, blood urea nitrogen (BUN), pneumonia severity index (PSI) class, body mass index (BMI), and albumin levels for prediction of 30-day mortality of patients older than 75 years

Though PCT was significantly associated with 30-day mortality in both of the younger and elderly groups in univariate analysis, multivariate analysis revealed that PCT was not an independent prognostic factor in both groups. Multivariate analysis revealed that BMI and albumin remained significant as factors related to 30-day mortality in both groups. In addition, BUN in the younger group and PSI in the elderly group were significant prognostic factors, respectively (Tables 2 and 3). According to ROC curve analysis, albumin and PSI, and albumin, BMI, and PSI revealed significantly wide AUC values compared to that of PCT in the younger and elderly groups, respectively (Figs 1 and 2).

**Table 2:** Univariate and multivariate analyses of factors related to 30-day mortality of patients younger than 74 years-old

		Univariate Logistic Regression		Multivariate Logistic		Regression
Variable	OR	95%CI	*P* value	OR	95%CI	*P* value
Age (years)	1.07	0.98–1.17	0.11			
Sex^a^	1.41	0.36–5.46	0.62			
Category of pneumonia^b^	4.08	1.20–13.93	0.02^c^	1.05	0.09-12.71	0.97
PSI class	2.72	1.40–5.29	< 0.01^c^	1.29	0.39-4.29	0.68
BMI (kg/m^2^)	0.58	0.42–0.79	< 0.01^c^	0.46	0.27-0.80	< 0.01^c^
Albumin (g/dL)	0.07	0.02–0.25	< 0.01^c^	0.05	0.00-0.73	0.03^c^
PCT (ng/mL)	1.07	1.02–1.13	0.01^c^	1.05	0.98-1.12	0.19
CRP (mg/dL)	1.07	1.02–1.14	0.01^c^	0.93	0.83-1.05	0.24
WBC(/mm^3^)	1.00	0.99–1.00	0.44			
BUN(mg/dL)	1.07	1.03–1.11	< 0.01^c^	1.08	1.00-1.17	0.04^c^
CCI	1.50	0.92-2.42	0.10			

*OR* Odds ratio, *Cl* Confidence interval, *RSI* Pneumonia severity index, *BMI* Body mass index, *PCT* Procalcitonin, *CRP* C-reactive protein, *WBC* White blood cell, *BUN* Blood urea nitrogen, *CCI* Charlson comorbidity index

^a^ Female vs male

^b^ Healthcare-associated pneumonia vs community acquired pneumonia

^c^Statistically significant

**Table 3:** Univariate and multivariate analyses of factors related to 30-day mortality of patients older than 75 years-old

	Univariate Logistic Regression			Multivariate Logistic Regression		
Variable	OR	95%CI	*P* value	OR	95%CI	*P* value
Age (years)	1.05	1.00–1.10	0.04^c^	1.02	0.96-1.08	0.60
Sex^a^	1.27	0.70–2.32	0.43			
Category of pneumonia^b^	2.14	1.18–3.86	0.01^c^	0.86	0.38-1.95	0.71
PSI class	4.24	2.59–6.94	< 0.01^c^	2.02	1.05-3.88	0.04^c^
BMI (kg/m^2^)	0.80	0.73–0.88	< 0.01^c^	0.84	0.75-0.94	< 0.01^c^
Albumin (g/dL)	0.38	0.26–0.54	< 0.01^c^	0.31	0.14-0.66	< 0.01^c^
PCT (ng/mL)	1.02	1.00–1.03	0.04^c^	1.01	0.98-1.03	0.62
CRP (mg/dL)	1.04	1.00–1.07	0.02^c^	1.01	0.97-1.06	0.56
WBC(/mm^3^)	1.00	1.00–1.00	0.29			
BUN(mg/dL)	1.04	1.02–1.05	< 0.01^c^	1.01	0.99-1.03	0.24
CCI	1.27	1.00–1.62	0.04^c^	1.07	0.77-1.48	0.69

*OR* Odds ratio, *Cl* Confidence interval, *PSI* Pneumonia severity index, *BMI* Body mass index, *PCT* Procalcitonin, *CRP* C-reactive protein, *WBC* White blood cell, *BUN* Blood urea nitrogen, *CCI* Charlson comorbidity index

^a^ Female vs male

^b^ Healthcare-associated pneumonia vs community acquired pneumonia

^c^Statistically significant

### Relationship between pneumonia severity and PCT, CRP, and WBC

**Fig. 3:**
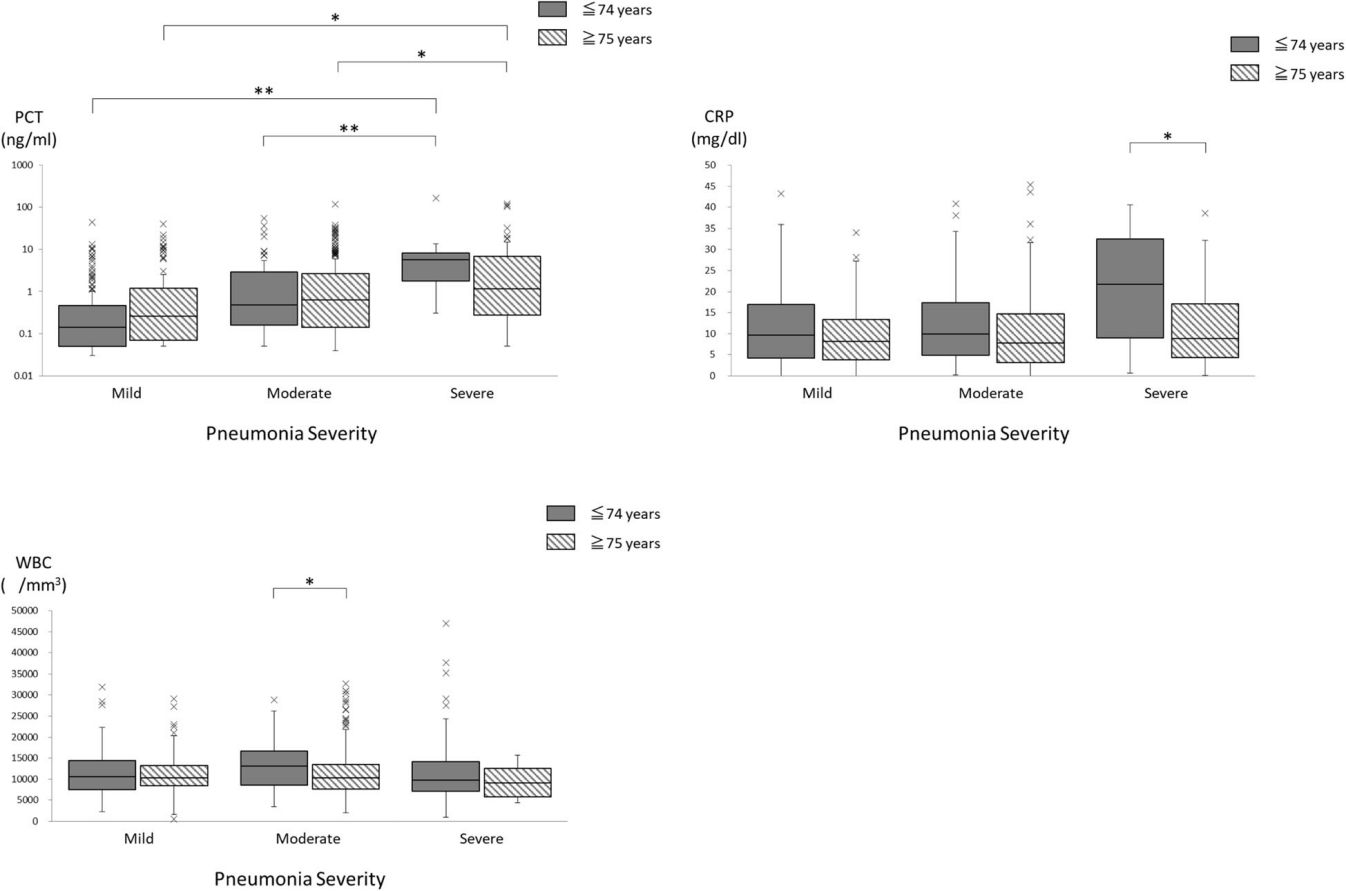
Box plots showing procalcitonin (PCT) levels, C-reactive protein (CRP) levels, and white blood cell (WBC) counts according to pneumonia severity determined by the pneumonia severity index of younger (≤74 years old) and elderly (≥75 years old) patients with pneumonia occurring outside the hospital setting. The box plots show 25th, 50th, and 75th percentiles, and outliers. *: *p* < 0.05, **: *p* < 0.01

Median (1st quartile, 3rd quartile) PCT levels in younger and elderly groups were 0.14 (0.05, 0.47) ng/mL and 0.26 (0.07, 1.19) ng/mL (*p* = 0.38) for mild pneumonia, 0.48 (0.16, 2.89) ng/mL and 0.63 (0.14, 2.63) ng/mL (*p* = 0.95) for moderate pneumonia, and 5.65 (1.78, 8.24) ng/mL and 1.15 (0.28, 6.81) ng/mL (*p* = 0.17) for severe pneumonia, respectively (Fig. 3, left upper). Median PCT levels of moderate and severe pneumonia increased by 229 and 3614%, respectively, compared with those of mild pneumonia in younger patients. Similarly, median PCT levels of moderate and severe pneumonia increased by 135 and 327%, respectively, compared with those of mild pneumonia in elderly patients. PCT levels significantly differed by pneumonia severity in both younger (*p* < 0.001) and elderly (p < 0.001) groups, and increased with increasing severity in both groups.

Median (1st quartile, 3rd quartile) CRP levels in younger and elderly groups were 9.66 (4.26, 16.99) mg/dL and 8.19 (3.84, 13.34) mg/dL (*p* = 0.26) for mild pneumonia, 9.88 (4.87, 17.42) mg/dL and 7.83 (3.15, 14.70) mg/dL (*p* = 0.08) for moderate pneumonia, and 21.71 (9.05, 32.51) mg/dL and 8.81 (4.37, 17.05) mg/dL (*p* = 0.02) for severe pneumonia, respectively (Fig. 3, right upper). Median CRP levels of moderate and severe pneumonia increased by 1.6 and 74.9%, respectively, compared with those of mild pneumonia in younger patients. Similarly, median CRP levels of moderate and severe pneumonia increased by − 1.5 and 9.5%, respectively, compared with those of mild pneumonia in elderly patients. CRP levels did not significantly differ by pneumonia severity in both younger (p = 0.08) and elderly (*p* = 0.59) groups.

Median (1st quartile, 3rd quartile) WBC counts in younger and elderly groups were 10,550 (7575, 14,400) /mm^3^ and 10,300 (8528, 13,325) /mm^3^ (p = 0.59) for mild pneumonia, 13,200 (8625, 16,700) and 10,400 (7700, 13,525) (*p* = 0.04) for moderate pneumonia, and 9200 (5800, 12,600) /mm^3^ and 9750 (7125, 14,250) /mm^3^ (*p* = 0.38) for severe pneumonia, respectively (Fig. 3, left lower). WBC counts did not significantly differ by pneumonia severity in the elderly group (*p* = 0.74), but did differ by severity in the younger group (p = 0.04), although the counts did not increase with increasing severity.

**Fig. 4:**
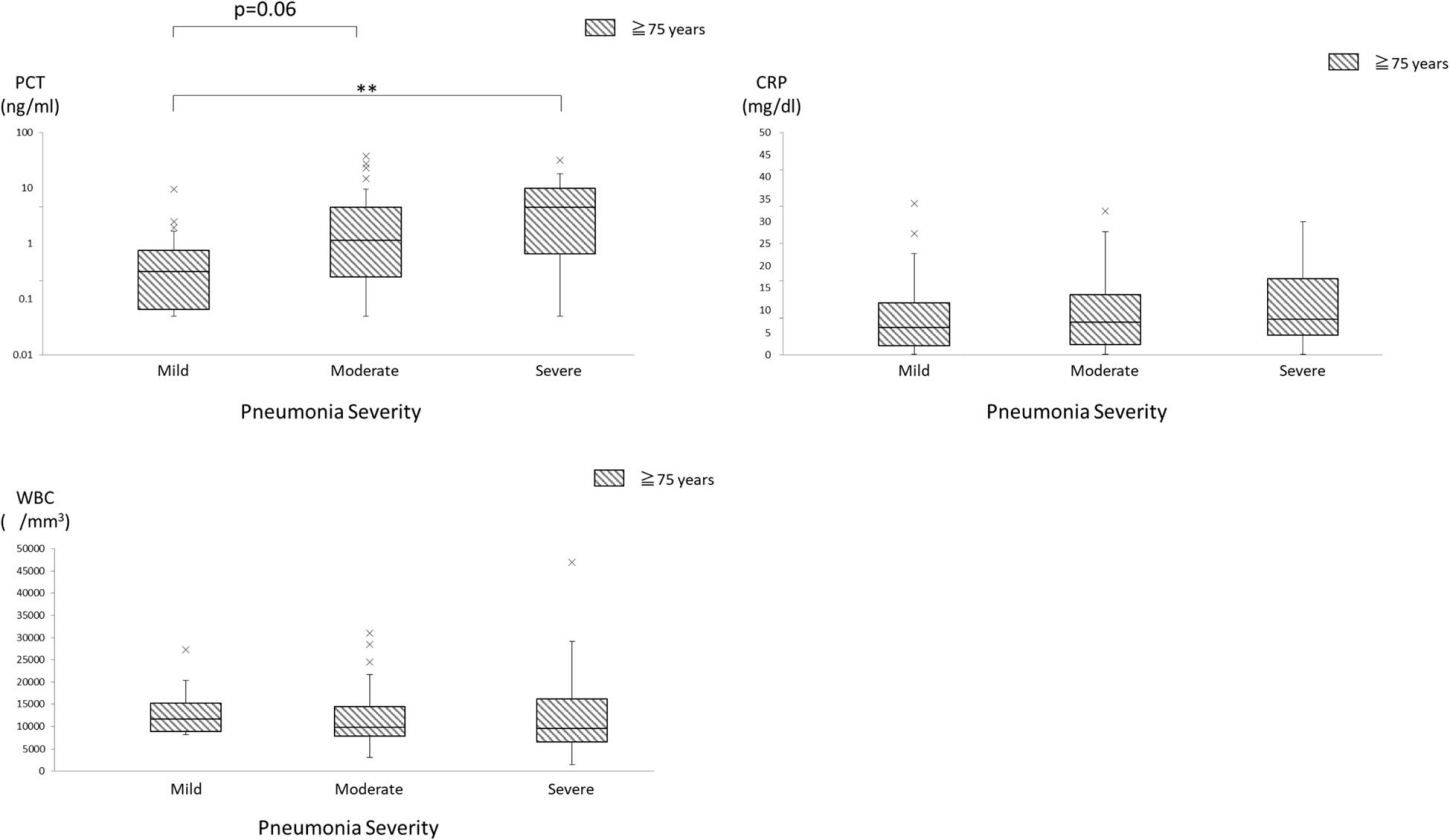
Box plots showing procalcitonin (PCT) levels, C-reactive protein (CRP) levels, and white blood cell (WBC) counts according to pneumonia severity determined by the pneumonia severity index of elderly (≥75 years old) patients with *Streptcoccus pneumoniae* pneumonia occurring outside the hospital setting. The box plots show 25th, 50th, and 75th percentiles, and outliers. **: *p* < 0.01

Next, we investigated the relationship between pneumonia severity and PCT levels, CRP levels, and WBC counts in elderly patients in which pneumonia was caused by *S. pneumoniae*, the most common pathogen identified in the study. Of those with mild, moderate, and severe pneumonia, 21 (19.1%), 42 (18.4%) and 24 (24.5%) patients had *S. pneumoniae* pneumonia, respectively. PCT levels significantly differed by severity (*p* = 0.03) and increased with increasing severity. (Fig. 4, left upper). CRP levels (*p* = 0.68) and WBC counts (*p* = 0.31) did not significantly differ by severity (Fig. 4, right upper and left lower).

## Discussion

PCT has been used to differentiate between infectious and noninfectious inflammatory respiratory diseases, or bacterial and non-bacterial respiratory infectious diseases [2–4]. Many investigators have reported the clinical usefulness of PCT in patients with CAP [5–7, 17–26], HCAP [37, 38], ventilator-associated pneumonia (VAP) [39–43], and hospital-acquired pneumonia (HAP) [44]. Although these studies included many elderly patients, the clinical usefulness of PCT in those patients was not consistent. Advanced age is associated with a decline in immune function, a phenomenon commonly referred to as immune senescence [45]. Immune senescence is generally characterized by chronic, low-grade, systemic inflammation and impaired responses to immune challenge. In elderly patients, complex immunologic rearrangements with chronic exposure to a variety of antigens may promote a subclinical inflammatory status defined as inflammaging, which can result in impaired PCT release in response to antigens [46]. It is currently unclear how the increased inflammation and declined functional reserve of parenchymal cells in elderly people would affect PCT release. In addition, the etiology of pneumonia may also be associated with different cytokine activation patterns. Thus, elderly patients with pneumonia might exhibit poor PCT elevation compared to their younger counterparts.

The geriatric population represents the fastest growing segment of the general population. Therefore, the clinical significance of PCT as diagnostic and prognostic factors in elderly patients is an important issue. While some investigators reported usefulness of PCT for diagnosing bacterial infection in the elderly [8, 11, 47], others revealed that PCT was inferior to CRP for predicting bacterial infection in these population [9, 48, 49]. As for predicting capacity for clinical course, Shi et al. reported that there were no differences in PCT between the improved and not-improved subjects in nosocomial pneumonia in the elderly [50]. To our knowledge, this is the first study to examine the relationship between PCT levels by pneumonia severity in younger and elderly patients with pneumonia, particularly patients with *S. pneumoniae* pneumonia, occurring outside the hospital setting. In addition, the present study included more elderly patients than those that previously assessed the clinical usefulness of PCT in the elderly population [8–11, 47–50]. We assessed the relationship between PCT and PSI, because mortality in this frail elderly population is frequently not directly due to pneumonia [51], and PSI could be more specific of pneumonia severity than all cause mortality. We found that PCT levels, but not CRP levels, significantly increased with increasing severity of pneumonia in both younger and elderly patients, although the degree of increase tended to be smaller in elderly patients under conditions of same severity. Kim et al. also reported the usefulness of PCT for predicting the severity of CAP in elderly patients, although they defined elderly as > 65 years of age, the sample size was small, and they did not analyze pneumonia caused by a specific pathogen [10]. The present study revealed that PCT increased with increasing severity of pneumonia even in patients older than 75 years, and also those with *S. pneumoniae* pneumonia.

Elderly patients, although representing the large majority of cases of pneumonia admitted to acute-care hospital wards, are often excluded from clinical studies due to the possible presence of multiple confounders. Elderly patients included in the present study also had many comorbidities. While Charlson comorbidity index was significantly associated with 30-day mortality in elderly patients in univariate analysis, it did not remain an independent prognostic factor in these patients in multivariate analysis.

Though many investigators have studied the utility of PCT in predicting mortality in various types of pneumonia including CAP [5–7, 17–26], HCAP [37, 38], VAP [39–43] and HAP [44], their results were not consistent While some reported PCT to be a reliable predictor for mortality [17–26], others showed that PCT was inferior to other markers such as PSI, CURB-65 [52], CRP, IL-6 [53], or BUN/albumin ratio [54] for predicting mortality. Several investigators reported that serial measurements of PCT were useful for predicting mortality [21, 50, 55]. In the present study, PCT did not remain an independent prognostic factor in the younger and elderly patients in our multivariate analysis. Albumin, BMI and PSI were independent prognostic factors, and superior to PCT for predicting 30-day mortality in the elderly patients. PCT is considered an inflammation marker like CRP and WBC, although more specific to bacterial infections compared to the latter two. In the present study, none of the inflammation markers assessed remained significant prognostic factors in multivariate analysis. This suggests that inflammation markers might not be independent prognostic markers when parameters known to influence prognosis, e.g., albumin or BMI, are comprehensively analyzed in patients with pneumonia occurring outside the hospital setting. Previous studies did not properly adjust for albumin or BMI in their multivariate analyses [6, 17–26].

The definition of the elderly varies according to the aim and design of research studies. Although the definition of elderly in previous studies that have investigated PCT ranged from 65 to 85 years, many have adopted the definition of > 75 years. The average age of the overall population in the present study was 76 years. Based on this, we defined the elderly as patients older than 75 years.

This study has some limitations worth noting. First, the study had a retrospective design and was conducted in a single hospital. A prospective cohort study involving multiple hospitals will be needed to confirm our results. Second, the number of younger patients with severe pneumonia was small. However, the number of such patients is naturally small, and several thousand pneumonia patients may be needed to obtain a sufficient number of younger patients with severe pneumonia. Thirdly, brain natriuretic peptide (BNP) or N-terminal-proBNP (NT-proBNP), which has been reported to be a prognostic marker of pneumonia [32, 33], was not included in our study, because these markers were measured in only about half of patients included in the study. Prognostic significance of these peptides in elderly patients with pneumonia needs to be investigated. Finally, analysis of PCT levels in bacteremia patients was not performed in the present study, because blood culture was performed in only 60% of patients, and bacteria grew in only 8 patients.

## Conclusions

PCT was not an independent predictor of 30-day mortality in both of elderly and younger patients with pneumonia acquired outside the hospital. PCT levels, but not CRP levels, significantly increased with increasing pneumonia severity in younger and elderly patients, although the degree of increase tended to be lower in elderly patients compared to younger patients for the same severity. PCT levels also significantly increased with increasing pneumonia severity in elderly patients with *Streptococcus pneumoniae* pneumonia.

## Abbreviations

AUC: Area under the curve
BMI: Body mass index
CAP: Community-acquired pneumonia
CI: Confidence interval
CRP: C-reactive protein
HCAP: Healthcare-associated pneumonia
MRSA: Methicillin-resistant *Staphylococcus aureus*
MSSA: Methicillin-sensitive *Staphylococcus aureus*
OR: Odds ratio
PCT: Procalcitonin
PSI: Pneumonia severity index
ROC: Receiver operating characteristic
WBC: White blood cell

## References

[1] Mandell LA, Wunderink RG, Anzueto A, Bartlett JG, Campbell GD, Dean NC (2007). Infectious Diseases Society of America / American Thoracic Society consensus guidelines on the management of community-acquired pneumonia in adults. Clin Infect Dis.

[2] Limper M, de Kruif MD, Duits AJ, Brandjes DPM, van Gorp ECM (2010). The diagnostic role of procalcitonin and other biomarkers in discriminating infectious from non-infectious fever. J Inf Secur.

[3] Kolditz M, Halank M, Schulte-Hubbert B, Hӧffken G (2012). Procalcitonin improves the differentiation between infectious and cryptogenic / secondary organizing pneumonia. J Inf Secur.

[4] Takeda S, Nagata N, Miyazaki H, Akagi T, Harada T, Kodama S (2015). Clinical utility of procalcitonin for differentiating between cryptogenic organizing pneumonia and community-acquired pneumonia. Int J Clin Med.

[5] Johansson N, Kalin M, Backman-Johansson C, Larsson A, Nilsson K, Hedlund J (2014). Procalcitonin levels in community-acquired pneumonia – correlation with aetiology and severity. Scand J Infect Dis.

[6] Masia M, Gutierrez F, Shum C, Padilla S, Navarro JC, Flores E (2005). Usefulness of procalcitonin levels in community-acquired pneumonia according to the patients outcome research team pneumonia severity index. Chest.

[7] Schuetz P, Wirz Y, Sager R, Christ-Crain M, Stolz D, Tamm M (2018). Effect of procalcitonin-guided antibiotic treatment on mortality in acute respiratory infections: a patient level meta-analysis. Lancet Infect Dis.

[8] Zhang H, Wang X, Zhang Q, Xia Y, Liu D (2017). Comparison of procalcitonin and high-sensitivity C-reactive protein for the diagnosis of sepsis and septic shock in the oldest old patients. BMC Geriatr.

[9] Nouvenne A, Ticinesi A, Folesani G, Cerundolo N, Prati B, Morelli I (2016). The association of serum procalcitonin and high-sensitivity C-reactive protein with pneumonia in elderly multimorbid patients with respiratory symptoms: retrospective cohort study. BMC Geriatr.

[10] Kim JH, Seo JW, Mok JH, Kim MH, Cho WH, Lee K (2013). Usefulness of plasma procalcitonin to predict severity in elderly patients with community-acquired pneumonia. Tuberc Respir Dis.

[11] Wang Y, Zhang S, Li L, Xie J (2018). The usefulness of serum procalcitonin, C-reactive protein, soluble triggering receptor expressed on myeloid cells 1 and clinical pulmonary infection score for evaluation of severity and prognosis of community-acquired pneumonia in elderly patients. Arch Gerontol Geriatr.

[12] Fine MJ, Auble TE, Yealy DM, Hanusa BH, Weissfeld LA, Singer DE (1997). A prediction rule to identify low-risk patients with community-acquired pneumonia. N Engl J Med.

[13] Chalmers JD, Singanayagam A, Akram AR, Mandal P, Short PM, Choudhury G (2010). Severity assessment tools for predicting mortality in hospitalised patients with community-acquired pneumonia. Systematic review and meta-analysis. Thorax.

[14] Loke YK, Kwok CS, Nirubsn A, Myint PK (2010). Value of severity scales in predicting mortality from community-acquired pneumonia: systematic review and meta-analysis. Thorax.

[15] Carrabba M, Zarantonello M, Bonara P, Hu C, Minonzio F, Cortinovis I (2012). Severity assessment of healthcare-associated pneumonia and pneumonia in immunosuppression. Eur Respir J.

[16] Jeong BH, Koh WJ, Yoo H, Um SW, Suh GY, Chung MP (2013). Performances of prognostic scoring systems in patients with healthcare-associated pneumonia. Clin Infect Dis.

[17] Boussekey N, Leroy O, Georges H, Devos P, d’Escrivan T, Guery B (2005). Diagnostic and prognostic values of admission procalcitonin levels in community-acquired pneumonia in an intensive care unit. Infection.

[18] Kruger S, Ewig S, Marre R, Papassotiriou J, Richter K, von Baum H (2008). Procalcitonin predicts patients at low risk of death from community-acquired pneumonia across all CRB-65 classes. Eur Respir J.

[19] Tseng JS, Chan MC, Hsu JY, Kuo BI, Wu CL (2008). Procalcitonin is a valuable prognostic marker in ARDS caused by community-acquired pneumonia. Respirology.

[20] Huang DT, Weissfeld LA, Kellum JA, Yealy DM, Kong L, Martino M (2008). Risk prediction with procalcitonin and clinical rules in community-acquired pneumonia. Ann Emerg Med.

[21] Schuetz P, Suter-Widmer I, Chaudri A, Christ-Crain M, Zimmerli W, Mueller B (2011). Prognostic value of procalcitonin in community-acquired pneumonia. Eur Respir J.

[22] Lacoma A, Rodrigues N, Prat C, Ruiz-Manzano J, Andreo F, Ramirez A (2012). Usefulness of consecutive biomarkers measurement in the management of community-acquired pneumonia. Eur J Clin Microbiol Infect Dis.

[23] Haeuptle J, Zaborsky R, Fiumefreddo R, Trampuz A, Steffen I, Frei R (2009). Prognostic value of procalcitonin in Legionella pneumonia. Eur J Clin Microbiol Infect Dis.

[24] Park JH, Wee JH, Choi SP, Oh SH (2012). The value of procalcitonin levels in community-acquired pneumonia in the ED. Am J Emerg Med.

[25] Andrijevic I, Matijasevic J, Andrijevic L, Kovacevic T, Zaric B (2014). Interleukin-6 and procalcitonin as biomarkers in mortality prediction of hospitalized patients with community acquired pneumonia. Ann Thorac Med.

[26] Kim MW, Lim JY, Oh SH (2017). Mortality prediction using serum biomarkers and various clinical risk scales in community-acquired pneumonia. Scand J Clin Lab Invest.

[27] LaCroix AZ, Lipson S, Miles TP, White L (1989). Prospective study of pneumonia hospitalization and mortality of U.S. older people: the role of chronic conditions, health behaviors, and nutritional status. Public Health Rep.

[28] Lee JH, Kim J, Kim K, Hwan Y, Rhee J, Kim TY (2011). Albumin and C-reactive protein have prognostic significance in patients with community-acquired pneumonia. J Crit Care.

[29] Viasus D, Garcia-Vidal C, Simonetti A, Manresa F, Dorca J, Gudiol F (2013). Prognostic value of serum albumin levels in hospitalized adults with community-acquired pneumonia. J Inf Secur.

[30] Ito A, Ishida T, Tokumasu H, Washio Y, Yamazaki A, Ito Y (2017). Prognostic factors in hospitalized community-acquired pneumonia: a retrospective study of a prospective observational cohort. BMC Pul Med.

[31] Falagas ME, Athanasoulia AP, Peppas G, Karageorgopoulos DE (2009). Effect of body mass index on the outcome of infections: a systemic review. Obes Rev.

[32] Christ-Crain M, Breidthardt T, Stolz D, Zobrist K, Bingisser R, Biedinger D (2008). Use of B-type natriuretic peptide in the risk stratification of community-acquired pneumonia. J Intern Med.

[33] Nowak A, Breidthardt T, Christ-Crain M, Bingisser R, Meune C, Tanglay Y (2012). Direct comparison of three natriuretic peptides for prediction of short- and long-term mortality in patients with community-acquired pneumonia. Chest.

[34] Miyazaki H, Nagata N, Akagi T, Takeda S, Harada T, Ushijima S (2018). Comprehensive analysis of prognostic factors in hospitalized patients with pneumonia occurring outside hospital: serum albumin is not less important than pneumonia severity assessment scale. J Infect Chemother.

[35] Hospital-acquired pneumonia guideline committee of the American Thoracic Society and Infectious Diseases Society of America (2005). Guidelines for management of adults with hospital-acquired pneumonia, ventilator-associated pneumonia, and healthcare-associated pneumonia. Am J Respir Crit Care Med.

[36] Charlson ME, Pompei P, Ales KL, MacKenzie CR (1987). A new method of classifying prognostic comorbidity in longitudinal studies: development and validation. J Chronic Dis.

[37] Porfyridis I, Georgiadis G, Vogazianos P, Mitis G, Georgiou A (2014). C-reactive protein, procalcitonin, clinical pulmonary infection score, and pneumonia severity scores in nursing home acquired pneumonia. Respir Care.

[38] Hong DY, Park SO, Kim JW, Lee KR, Baek KJ, Na JU (2016). Serum procalcitonin: an independent predictor of clinical outcome in health care-associated pneumonia. Respiration.

[39] Duflo F, Debon R, Monneret G, Bienvenu J, Chassard D, Allaouchiche B (2001). Alveolar and serum procalcitonin: diagnostic and prognostic value in ventilator-associated pneumonia. Anesthesiology.

[40] Luyt CE, Guerin V, Combes A, Trouillet JL, Ayed SB, Bernard M (2005). Procalcitonin kinetics as a prognostic marker of ventilator-associated pneumonia. Am J Respir Crit Care Med.

[41] Ji B, Zhao X, Li S (2015). Serum procalcitonin level and mortality risk in critically ill patients with ventilator-associated pneumonia. Cell Physiol Biochem.

[42] Seligman R, Seligman BG, Teixeira PJ (2011). Comparing the accuracy of predictors of mortality in ventilator-associated pneumonia. J Bras Pneumol.

[43] Tanriverdi H, Tor MM, Kart L, Altin R, Atalay F, SumbSumbuloglu V (2015). Prognostic value of serum procalcitonin and C-reactive protein levels in critically ill patients who developed ventilator-associated pneumonia. Ann Thorac Med.

[44] Kumar S, Jan RA, Rasool R, Fomda BA, Koul PA, Shah S (2018). Utility of procalcitonin in predicting mortality among cases of hospital-acquired pneumonia: a north Indian study. Egypt J Chest Dis Tuberc.

[45] Goronzy JJ, Weyand CM (2013). Understanding immune senescence to improve vaccine responses. Nat Immunol.

[46] Franceschi C, Campisi J (2014). Chronic inflammation (inflammaging) and its potential contribution to age-associated diseases. J Gerontol A Biol Sci MedSci.

[47] Gomez-Cerquera JM, Daroca-Perez R, Baeza-Trinidad R, Casanas-Martinez M, Mosquera-Lozano JD, Ramalle-Gomara E (2015). Validity of procalcitonin for the diagnosis of bacterial infection in elderly patients. Enferm Infecc Microbiol Clin.

[48] Stucker F, Herrmann F, Graf JD, Michel JP, Krause KH, Gavazzi G (2005). Procalcitonin and infection in elderly patients. J Am Geriatr Soc.

[49] Yan L, Liao P, Xu LL, Zhao Y (2014). Usefulness of procalcitonin in elderly patients with bacterial infection. Clin Lab.

[50] Shi Y, Xu Y-c, Rui X, H-m Z, Wang Y, Du W (2014). Procalcitonin kenetics and nosocomial pneumonia in older patients. Respir Care.

[51] Mortensen EM, Coley CM, Singer DE, Marrie TJ, Obrosky DS, Kapoor WN (2002). Causes of death for patients with community-acquired pneumonia: results from the pneumonia patient outcomes research team study. Arch Intern Med.

[52] Zhang ZX, Zhang W, Liu P, Yang Y, Tan WC, Ng HS (2016). Prognostic value of pneumonia severity index, CURB-65, CRB-65, and procalcitonin in community-acquired pneumonia in Singapore. Pcoceedings of Singapore Healthcare.

[53] Menendez R, Martinez R, Reyer S, Mensa J, Filella X, Marcos MA (2009). Biomarkers improve mortality prediction vy prognostic scales in community-acquired pneumonia. Thorax.

[54] Ugajin M, Yamaki K, Hirasawa N, Yagi T (2014). Predictive values of semi-quantitative procalcitonin test and common biomarkers for the clinical outcomes of community-acquired pneumonia. Respir Care.

[55] McCluskey SM, Schuetz P, Abers M, Bearnot B, Morales M, Hoffman D (2017). Serial procalcitonin as a predictor of bacteremia and need for intensive care unit care in adults with pneumoniacitonin as a predictor of bacteremia and need for intensive care unit care in adults with pneumonia, including those with highest severity: a prospective cohort study. Open Forum Infect Dis.

